# Plasmalogen‐rich foods promote the formation of cubic membranes in amoeba *Chaos* under stress conditions

**DOI:** 10.1002/2211-5463.13241

**Published:** 2021-07-19

**Authors:** Ketpin Chong, Zakaria A. Almsherqi, Ruijiang Zhuo, Yuru Deng

**Affiliations:** ^1^ Department of Physiology Yong Loo Lin School of Medicine National University of Singapore Singapore; ^2^ School of Ophthalmology and Optometry Eye Hospital School of Biomedical Engineering Wenzhou Medical University China; ^3^ Wenzhou Institute University of Chinese Academy of Sciences Wenzhou China

**Keywords:** cell protection, cubic membrane, plasmalogen, starvation stress

## Abstract

Previous studies have indicated that the ability to form cubic membrane (CM), a three‐dimensional periodic structure with cubic symmetry, in amoeba (*Chaos carolinense*) under stress conditions depends on the type of food organism supplied before cell starvation. The significant increase in docosapentaenoic acid (DPA; C22:5n‐6) during the starvation period has been reported to induce CM formation and support *Chaos* cell survival. In this article, we further investigated the lipid profiles of food organisms of the *Chaos* cells to reveal the key lipid components that might promote CM formation. Our results show that the lipids extracted from cells of the native food organism *Paramecium multimicronucleatum* are enriched in plasmalogens. More specifically, plasmalogen phosphatidylcholine and plasmalogen phosphatidylethanolamine might be the key lipids that trigger CM formation in *Chaos* cells under starvation stress conditions. Unexpectedly, CM formation in these cells is not supported when the native food organism was replaced with plasmalogen‐deficit *Tetrahymena pyriformis* cells. Based on a previous lipidomics study on amoeba *Chaos* and this study on the lipid composition of its food organisms, three key lipids (plasmalogen phosphatidylcholine, plasmalogen phosphatidylethanolamine and diacyl‐phosphatidylinositol) were identified and used for liposomal construction. Our *in vitro* study revealed the potential role of these lipids in a nonlamellar phase transition. The negative staining transmission electron microscopy data of our liposomal constructs support the notion that plasmalogens may curve the membrane, which, in turn, may facilitate membrane fusion and vesicular formation, which is crucial for membrane dynamics and trafficking.

## Graphical abstract

Plasmalogens, abundant ether‐bonded phospholipids, are of significance in terms of both evolutionary history and their biological roles in membrane structure and dynamics. They are particularly enriched in the human nervous, immune, and cardiovascular systems, in addition to being present in primitive single‐celled amoeba. Plasmalogen preconditioning is required to trigger intracellular cubic membrane transition to further promote cell survival, supporting potential benefits of dietary plasmalogen therapy in stress‐associated disease prevention.

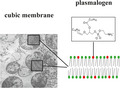

**Abbreviations**
CM: cubic membrane
diacyl‐PI: diacyl‐phosphatidylinositol
DOPC: 1,2‐dioleoyl‐sn‐glycero‐3‐phosphocholine
DOPE: 1,2‐dioleoyl‐sn‐glycero‐3‐phosphoethanolamine
DPA: docosapentaenoic acid
lysoPC: lysophosphatidylcholine
lysoPE: lysophosphatidylethanolamine
*Para*
: *Paramecium multimicronucleatum*
PC: phosphatidylcholine
PE: phosphatidylethanolamine
PI: phosphatidylinositol
pPC: plasmalogen phosphatidylcholine
pPE: plasmalogen phosphatidylethanolamine
TEM: transmission electron microscopy
*Tetra*
: *Tetrahymena pyriformis*

Approximately 20% of phospholipids are plasmalogens in human tissue, where they are particularly rich in the brain, heart and immune cells [1]. Plasmalogens are characterized with a unique vinyl–ether bond at sn‐1 and a regular ester bond at the sn‐2 position of the glycerol backbone, distinguishing them from their diacyl counterparts carrying ester bond at sn‐1 position instead [1]. Despite their abundance in multiple cell types, tissues and organs, the significance of plasmalogens in the biological system remains to be revealed.

The accumulated evidence suggests that plasmalogens may act as the first line of sacrificing molecules in preventing oxidative damage. Excessive reactive oxygen species and/or hydrogen peroxide produced in cells are able to react more readily with plasmalogens as opposed to reacting with other important biomolecules. This may thus prevent lipid peroxidation of the cell membrane and avoid further cell damage [2]. An emerging property of plasmalogens is to promote nonlamellar membrane transformation [3, 4, 5]. Glaser and Gross [6] reported that *in vitro* membrane vesicles with varying amounts of plasmalogens could induce different nonlamellar structure formation, suggesting the role of plasmalogens in facilitating membrane fusion. A recent structural study by Angelova *et al*. [7] further revealed that plasmalogens could strongly influence the membrane thickness and curvature. Deng *et al*. [8] reported that a significant increase in plasmalogen phosphatidylcholine (pPC) in amoeba *Chaos* cells under starvation stress is associated with nonlamellar cubic membrane (CM) formation. The higher level of pPC in amoeba *Chaos* cells was observed in *Paramecium multimicronucleatum* (*Para*)‐fed amoeba *Chaos* cells, but not in *Tetrahymena pyriformis* (*Tetra*)‐fed cells [8]. Interestingly, mitochondrial inner membrane rearrangement into cubic morphology was observed to occur when amoeba *Chaos* cells were fed with the extracted lipids from either *Para* or polyunsaturated fatty acids, specifically omega‐6 docosapentaenoic acid (DPA) [8]. Furthermore, it has been observed that liposome constructs using the extracted lipids from amoeba *Chaos* cells, which have been exclusively fed with *Para*, do induce cubic or hexagonal organization *in vitro* [8]. Based on the earlier observations, it has been proposed that plasmalogen might promote CMs formation, and plasmalogen‐rich CMs might act as a ‘protective’ shelter to minimize the oxidation of biologically essential macromolecules (lipids and RNAs) [9, 10, 11].

Based on the fact that the alterations of plasmalogen level and nonlamellar membrane transformation are linked both *in vitro* [3, 4, 5, 7] and *in vivo* [8], we speculate that there might be a correlation between plasmalogen availability as a result of food supply/deficiency and nonlamellar membrane formation. As such, we investigated the relation between plasmalogen levels of the food supply and nonlamellar membrane transformations using amoeba *Chaos* cells as an experimental model.

Our findings in the study of food supply of amoeba *Chaos* cells and CM formation under starvation and stressed conditions may help us to understand the molecular mechanism of nonlamellar membrane transformation, vesicle formation and vesicular fusion, especially at synaptic vesicles, where plasmalogens are abundant and essential for neuronal function [1, 12, 13].

## Materials and methods

### Cells and reagents

Amoeba *Chaos* and *Tetra* cells were purchased from Carolina Biological Supply Co. *Para* cells were a kind gift from Dr. Richard D. Allen’s laboratory [14] and Dr. Masaki Ishida’s laboratory (Nara University of Education, Japan). All other chemicals and reagents, unless mentioned otherwise, were purchased from Sigma‐Aldrich (St. Louis, MO, USA).

### *Amoeba* Chaos *mass culture*

The amoeba *Chaos* cell cultures were maintained in an inorganic amoeba medium containing 0.5 mm CaCl_2_, 0.05 mm MgSO_4_, 0.16 mm dipotassium phosphate and 0.11 mm potassium dihydrogen phosphate dissolved in MilliQ water [15]. Amoeba *Chaos* cells were kept in large treated Pyrex^®^ baking dishes filled with amoeba medium in darkness, at 22–24 °C on the benchtop. Amoeba *Chaos* cells were fed every 2–3 days only with 7‐day‐old *Para* or *Tetra* cultures at the late log or early stationary phase of growth [14, 16]. Both *Paramecium* and *Tetrahymena* cultures were maintained in growth media as previously described [16] and used for amoeba *Chaos* cell feeding. All Z‐4,7,10,13,16 DPAs (C22:5‐6; Sigma‐Aldrich Pte. Ltd., Singapore) were dissolved in diethyl ether and added to the amoeba *Chaos* cell culture medium at a final concentration of 100 μm. A total of 100 μm DPA and 100 μg·mL^−1^ extracted lipids from *Paramecium* were added to well‐fed amoeba cell cultures, respectively, according to the regular feeding schedule [16]. Amoeba *Chaos* cells were then fixed in 2.5% glutaraldehyde for further transmission electron microscopy (TEM) processing.

### *Amoeba* Chaos *cell harvest*

Before harvesting the amoeba *Chaos* cells, the cultures were gently washed several times with amoeba medium to remove the food organisms. Individual amoeba *Chaos* cells were then picked using a disposable Pasteur pipette into a glass beaker with amoeba medium, in which they were allowed to settle to the bottom simply by gravity. The supernatant was siphoned off, and the clean amoeba *Chaos* cells were ready for further processing. No food organisms (*Para* or *Tetra*) were added to amoeba *Chaos* cells culture for 7 days in starvation treatment to obtain the mitochondria with and without CM, respectively.

### Transmission electron microscopy

The amoeba *Chaos* cells [from 7‐day starved and fed amoeba *Chaos* cells (*Para/Tetra*) cultures] were primarily fixed with 2.5% glutaraldehyde (Agar Scientific) at 4 °C overnight followed by secondary fixation using 1% osmium tetroxide (OsO_4_) (Ted Pella, Inc., Redding, CA, USA) for 1 h at room temperature. Fixed samples were subjected to sequential dehydration by immersion in a graded series of ethanol dehydration steps. Preparations were embedded in Epon Resin (Pelco, Clovis, CA, USA) and sectioned with approximately 50–70 nm thickness using an ultramicrotome (Leica, Wetzlar, Germany) and stained in 3% uranyl acetate (Electron Microscopy Sciences, Hatfield, PA, USA) followed by Reynold's lead citrate. The ultrathin sections were viewed and examined under TEM (JEM1010; JEOL Ltd., Tokyo, Japan). For the negative staining TEM studies, 1% of lanthanum nitrate hexahydrate (Sigma) diluted in PBS buffer was used. Samples are air‐dried on a copper grid (Ted Pella, Inc.) and stained directly using gadolinium (III) acetate tetrahydrate (Sigma) before viewing under TEM.

### Lipid extraction

Lipids from *Para* or *Tetra* were extracted and analyzed for phospholipids, including plasmalogens. Phospholipids were extracted following Bligh and Dyer [17]. In brief, the cells were collected and spun down at 196 ***g*** with Multifunction benchtop centrifuge BR4i compact series (Jouan; Inov Solutions, Milford, MA, USA). The collected cell pellets were separately homogenized in chloroform/methanol (1 : 2, v/v), and the mixture was vortexed for 30 s. Lipids were further extracted at 4 °C under vigorous shaking for 10 min. A total of 0.3 mL chloroform and 0.3 mL deionized water (cold) were then added to the mixture and vortex to mix for 30 s followed by incubation on ice for 1 min. Phases were separated by high‐speed centrifugation at 6700 ***g*** for 2 min, and the lower phase was transferred to a fresh tube (extract 1). The residual aqueous phase and cell remnants were re‐extracted with 0.6 mL chloroform as described earlier, and the organic (lower) phase was obtained (extract 2). Extracts 1 and 2 were then combined and dried in a vacuum concentrator, SpeedVac (Thermo Savant, Milford, MA, USA), and stored at −20 °C. Before analysis, lipids were dissolved in chloroform/methanol (1 : 1, v/v).

### Analysis of lipids by mass spectrometry

Following a protocol adapted from Shui *et al*. [18], phospholipids and plasmalogens were quantified using shotgun‐tandem mass spectrometry (MS) approach. The lipids are first dissolved in chloroform/methanol (1 : 1, v/v) and mixed 1 : 1 with an internal standard solution. The internal standard solution was prepared for the lipid species: lysophosphatidylcholine (lysoPC) and phosphatidylcholine (PC) diluted in chloroform/methanol (1 : 1, v/v) to a total volume of 1 mL. The internal standard solution was prepared for the lipid species phosphatidylethanolamine (PE), phosphatidylserine (PS), phosphatidylglycerol (PG) and phosphatidylinositol (PI) diluted in chloroform/methanol (1 : 1, v/v) to a total volume of 6 mL. Samples were then introduced into the mass spectrometer using an Agilent 1200 high‐performance liquid chromatography system without chromatographic separation. The flow rate was 250 μL·min^−1^, and the analysis time was 1.7 min. The mass spectrometer was an Applied Biosystem Triple Quadrupole/Ion Trap mass spectrometer 4000 trap (Applied Biosystems, Foster City, CA, USA). The lipid species were quantified using multiple reaction monitoring and positive ionization lysoPC and PC or negative ionization PE, PS, PG and PI.

### Liposome preparation

Liposome preparation in this study follows the protocol from Avanti Polar Lipids Inc. (Alabaster, AL, USA) Synthetic lipids, 18 : 1 (Δ9‐Cis) PC 1,2‐dioleoyl‐sn‐glycero‐3‐phosphocholine (DOPC) and 18 : 1 (Δ9‐Cis) PE 1,2‐dioleoyl‐sn‐glycero‐3‐phosphoethanolamine (DOPE) (Avanti Polar Lipids Inc., AL, USA), were dissolved in chloroform. The solvent was then removed using a centrifugal evaporator (Jouan RC1022). This was followed by hydration of the lipid cake/film by adding deionized water and vigorous agitation for 1 h. At this point, large multilamellar vesicles were obtained. After the hydration was successfully completed, sizing of lipid suspension was performed to obtain small unilamellar vesicles via sonication for 15 min using a sonicator: ultrasonic bath (VWR International, Radnor, PA, USA) for TEM analysis. In this study, synthetic pPC, plasmalogen phosphatidylethanolamine (pPE) and/or diacyl‐phosphatidylinositol (diacyl‐PI) were added individually or in combination to verify their contribution to the membrane shape of liposomes.

## Results and Discussion

Nonlamellar CM formation induction under multiple stress conditions [19] in amoeba *Chaos* cells has disclosed the following potential roles of CM as (a) supporting cell survival under starvation and stressed conditions [20], and (b) a radical scavenging [11] and antioxidant defense system [10]. However, information on the detailed molecular mechanism of CM formation remains to be revealed.

### Nutrition determines CM formation and the fate of *Chaos* cell survival

**Fig. 1:**
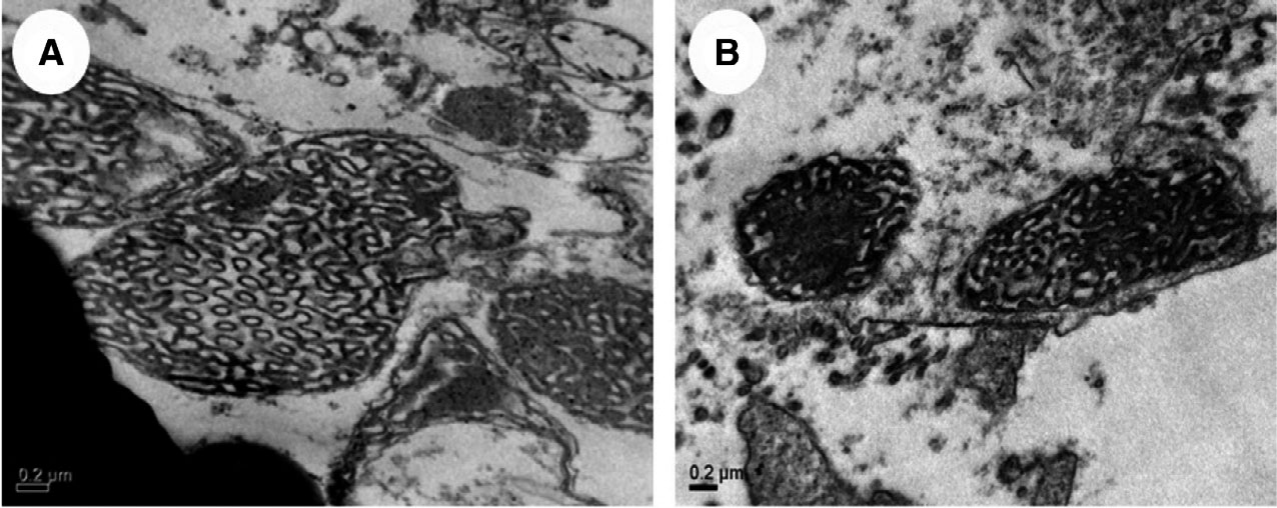
TEM micrographs of amoeba *Chaos* (*Tetra*) fed with extracted lipids from *Paramecium* and DPA. (A) Amoeba *Chaos* cells fed with 100 µg·mL^−1^ extracted lipids from *Paramecium* and (B) 100 µm DPA. Scale bars: 0.2 µm. Three independent experiments were performed [approximately 10 mitochondria of (A) and (B) were examined].

It was previously reported that amoeba *Chaos* cells with the presence of CM mitochondria appear to survive better under long‐term starvation and stress conditions [20]. CM formation induced in amoeba *Chaos* cells under starvation and stressed conditions is simply through food deprivation. Interestingly, in contrast with amoeba *Chaos* cells cultured and fed with *Paramecium multimicronucleatum* [hereinafter referred to as amoeba (*Para*)], amoeba *Chaos* cells cultured with *Tetra* as food organisms [hereinafter referred to as amoeba (*Tetra*)] could not induce mitochondrial CM formation in starvation and stress conditions [20]. In this report, amoeba *Chaos* cells fed with lipids extracted directly from the food organisms (Fig. 1) were indeed necessary to induce CM formation in amoeba *Chaos* cells in response to starvation and stressed conditions. From a previous report [20], CMs appeared and stayed in amoeba (*Para*) over a long course of starvation (up to 21 days). Hence it is plausible that CM formation requires certain exogenous nutrition as a prerequisite. Furthermore, the cell survival rate with the presence of CMs in amoeba (*Para*) is significantly higher than that of amoeba (*Tetra*) without CMs under starvation and stressed conditions. The data from a previous report [20] suggest that the possibility of nutrition (from *Paramecium*) being the determining factor of mitochondrial CM formation in response to starvation and stressed conditions and such nutrient element might be correlated to the improved amoeba *Chaos* cell survival.

### *Paramecium* lipids feeding endows the potential of CM formation in amoeba *Chaos*

It was previously reported that DPA (C22:5n‐6) is one of the key elements in CM formation in amoeba *Chaos* cells [8]. The authors fed polyunsaturated fatty acids (specifically omega‐6 DPA) in excess to well‐fed amoeba *Chaos* cell cultures (*Para*‐fed) and were able to induce CM formation without the application of any starvation stress. As such, it is intriguing to know whether lipids extracted from *Paramecium* are able to induce CM formation.

The extracted lipids from *Paramecium* were first fed to the control amoeba (*Tetra*). It should be emphasized that as opposed to amoeba (*Para*), CM was not inducible by starvation in amoeba (*Tetra*). Amoeba (*Tetra*) thus serves as a good control because it rules out the possibility of accidental CM induction under starvation stress. Also, more resounding effects of DPA and lipids extracted from *Paramecium* food organisms have been found on the potential formation of CMs.

Interestingly, the mitochondrial inner membranes of amoeba (*Tetra*) fed with extracted *Paramecium* lipids transformed into CMs (Fig. 1A). To validate whether DPA alone is sufficient for such induced membrane transformation in amoeba (*Tetra*), amoeba *Chaos* were also fed with DPA for a comparative study. DPA‐treated amoeba (*Tetra*) (Fig. 1B) rendered morphological changes in the mitochondrial membranes, but the cubic morphology was less prominent than those observed in *Paramecium* lipid‐treated amoeba (*Tetra*). Strikingly, lipids extracted from *Paramecium* appear to be the ‘prerequisite nutrients’ for CM induction under starvation stress in amoeba (*Tetra*), suggesting that dietary phospholipids supplementation might be sufficient and more superior compared with solely DPA fatty acids to induce any significant alteration of the membrane structure and function.

### *Paramecium* lipids carry a larger amount of plasmalogens compared with *Tetrahymena* lipids

Because the full crude extracted *Paramecium* lipids were able to induce CM formation in amoeba (*Tetra*) under starvation stress, the differences in content and composition of lipids between the two distinct food organisms of amoeba *Chaos* cells, namely, *Para* and *Tetra*, may help uncover the key nutrients (lipids) promoting CM formation in amoeba *Chaos* cells. Our previous study indicated that a sufficient amount of plasmalogens was present in amoeba *Chaos* cells to trigger CM formation under starvation stress conditions [8]. The unique vinyl–ether‐bonded plasmalogens have been reported to promote nonlamellar hexagonal and/or cubic phase transition *in vitro* [3, 4, 5, 7, 21]. As such, we logically speculate that plasmalogens might be crucial for CM formation *in vivo*. Thus, a lipid profile study was conducted.

**Fig. 2:**
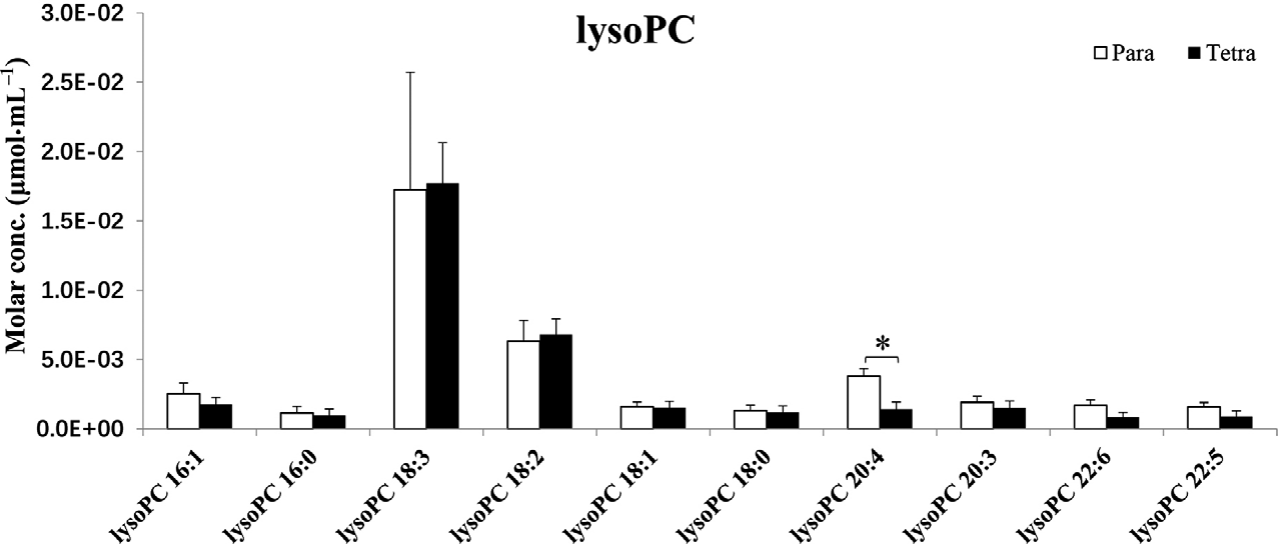
Bar graph representing lysolipid (lysoPC) levels compared between *Paramecium* and *Tetrahymena*. Data are means (± standard deviation) of three independent experiments. *P < 0.05 by Student’s *t*‐test. conc., concentration.

**Fig. 3:**
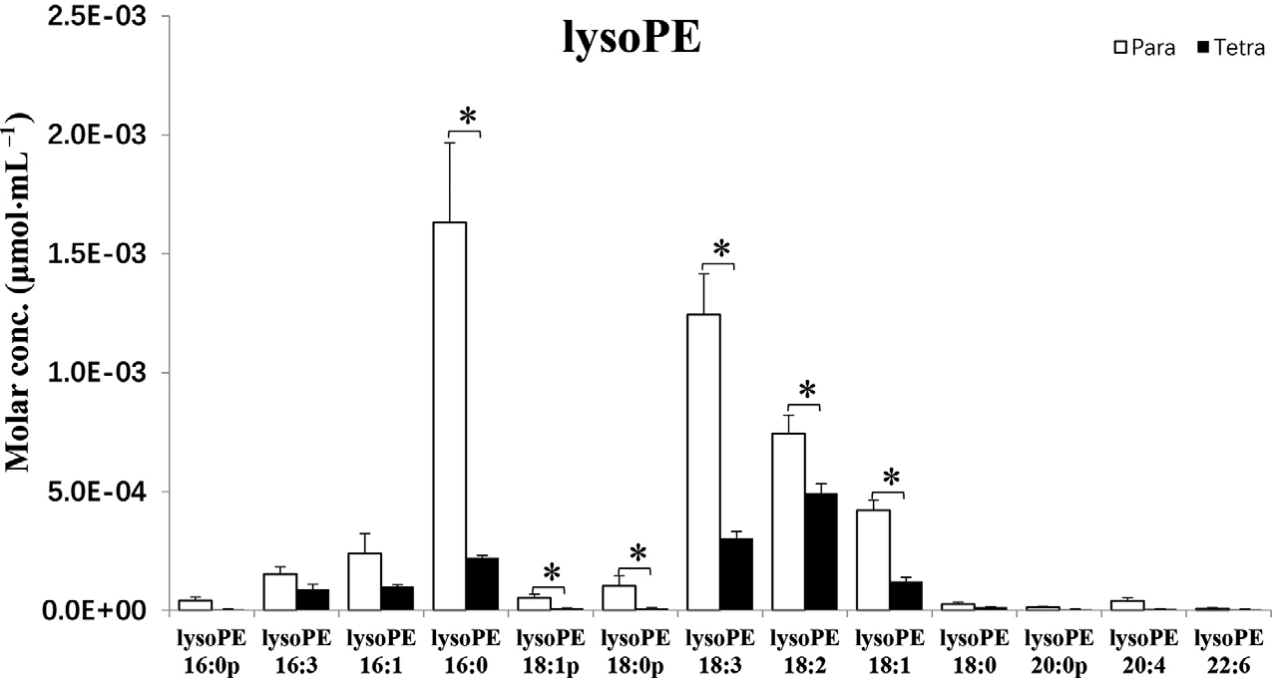
Bar graph representing lysolipid (lysoPE) and pPE levels compared between *Paramecium* and *Tetrahymena*. Data are means (± standard deviation) of three independent experiments. *P < 0.05 by Student’s *t*‐test. conc., concentration.

**Fig. 4:**
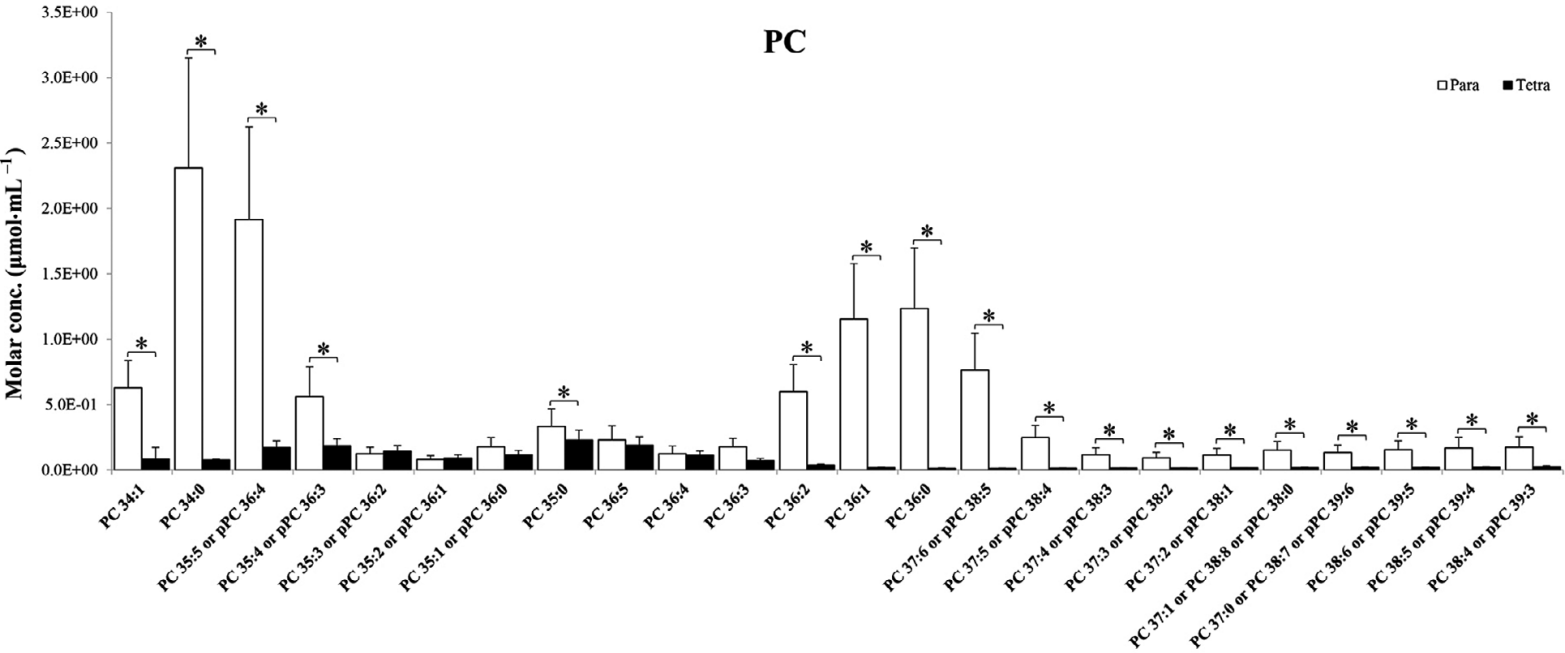
Bar graph representing PC and pPC levels compared between *Paramecium* and *Tetrahymena*. Data are means (± standard deviation) of three independent experiments. *P < 0.05 by Student’s *t*‐test. conc., concentration.

Figures 2 and 3 represent the differences in the lysophospholipids of *Paramecium* and *Tetrahymena*. Lysophospholipids are natural products formed by hydrolysis of phospholipids. The majority of lysoPCs elucidated are not significantly different between *Paramecium* and *Tetrahymena*, except for lysoPCs (20 : 4) (Fig. 2). In contrast, Fig. 3 shows certain lysophosphatidylethanolamines (lysoPEs) eluted that are significantly higher in *Paramecium* compared with *Tetrahymena*; they are, namely, lysoPE (C16:0p) (where p represents the presence of plasmenyl group), lysoPE (C16:0), lysoPE (C18:1p), lysoPE (C18:0p), lysoPE (C18:3), lysoPE (C18:2) and lysoPE (C18:1). Apparently, lysoPE (C16:0), lysoPE (C18:3), lysoPE (C18:2) and lysoPE (C18:1) are more abundant than the rest of lysoPEs revealed here. Notably, lysoPE species (C16:0p), (C18:1p) and (C18:0p) are plasmenyl lipids of PE (pPE). Although they are present in low amounts in both *Paramecium* and *Tetrahymena*, their relatively greater amounts in *Paramecium* are statistically different (*P < 0.05). Figure 4 shows a large number of PCs and pPCs that are significantly more abundant in *Paramecium* compared with *Tetrahymena*. However, some of the PCs and pPCs are in such contrastingly higher abundance that their presence might be paramount compared with the rest. Some examples are PC (34:1), PC (C34:0), PC (C36:2), PC (C36:1), PC (C36:0) and pPC (C36:4), pPC (C36:3), pPC (C38:5), pPC (C38:4).

The lipid data of food organisms of amoeba *Chaos* cells shown in Figs 2, 3, 4 revealed a significant difference in plasmalogen levels in these two food organisms (specifically, pPC is in relatively high abundance in *Paramecium* compared with *Tetrahymena*). The higher level of pPC in *Paramecium* lipid extract is consistent with the previous report on *Paramecium*‐fed amoeba *Chaos* cells under starvation and stressed conditions, with a significant increase of pPC [8].

**Fig. 5:**
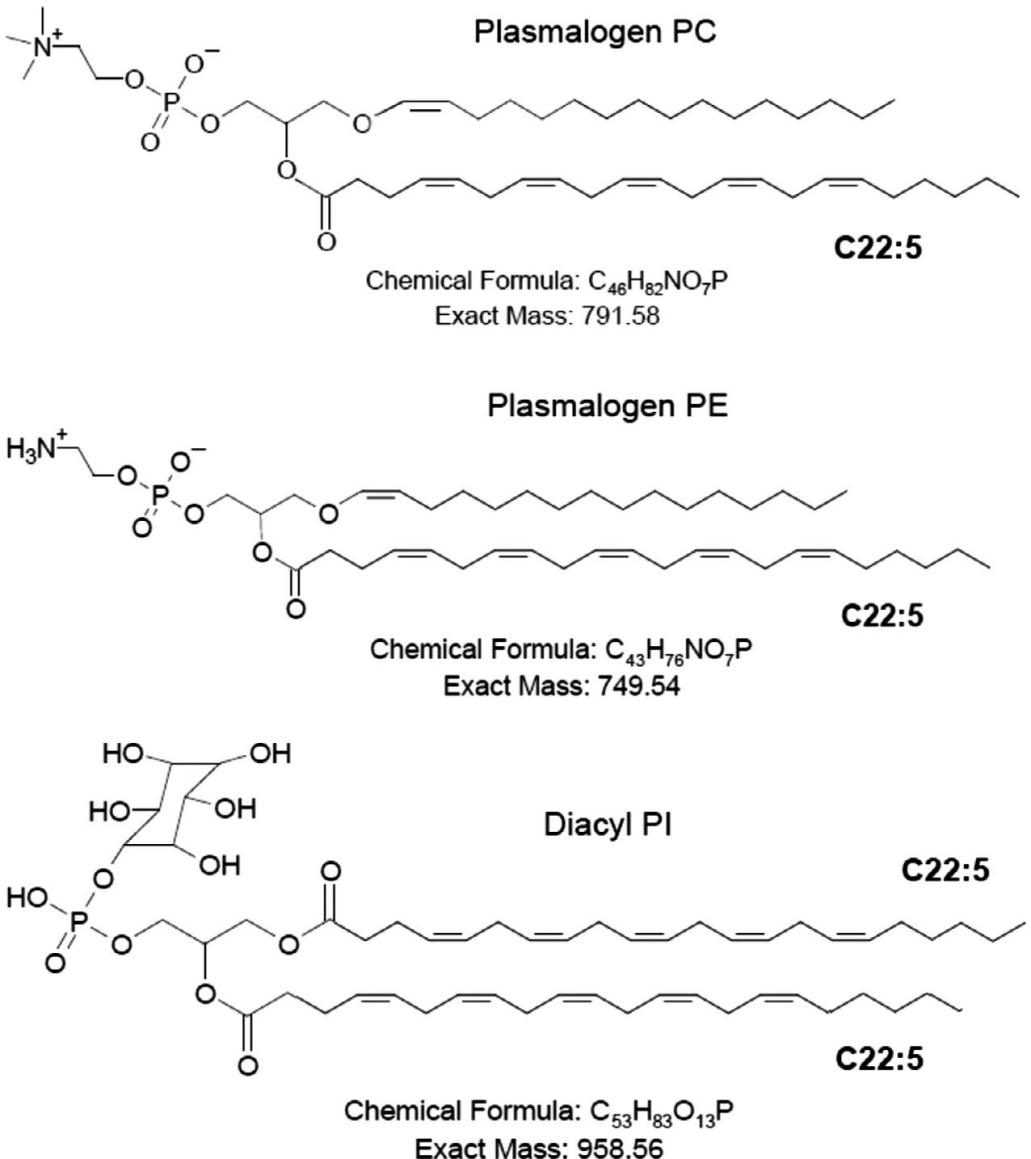
Chemical structures of three synthesized lipids custom‐made based on amoeba *Chaos* lipid data [8], namely, pPC, pPE and diacyl‐PI with unsaturated fatty acid chains C22:5n‐6.

Whether pPCs or pPEs are the key elements as preconditioning nutrients for amoeba *Chaos* cells to trigger CM formation remains to be uncovered. We thus further examined the effects of three synthesized custom‐made amoeba CM‐derived phospholipids (Fig. 5) on curving the membrane to promote nonlamellar membrane transformation *in vitro*.

### Synthesized plasmalogens may curve lipidic lamellar membrane phase *in vitro*

Deng *et al*. [8] reported that there was a significant increase in C22:5n‐6‐containing pPCs (C16:0p/C22:5n‐6) in amoeba *Chaos* (*Para*) on starvation when CM appeared. Although it was concluded that CM formation was attributed to DPA (C22:5n‐6), the significant increase in pPC (C16:0p/C22:5n‐6) levels observed in starved amoeba *Chaos* cells [8] might well prove the inadequacy of DPA alone in inducing CM formation compared with ‘plasmalogen‐rich’ extracted *Paramecium* lipids.

To validate the potency of plasmalogens on CM transformation, three major amoeba lipids, pPC (C16:0p/C22:5n‐6), pPE (C16:0p/C22:5n‐6) and diacyl‐PI (C22:5n‐6/C22:5n‐6) (Fig. 5), were custom‐made and purchased from Avanti Polar Lipids, Inc. based on our lipidomic data of amoeba *Chaos* cells [8]. DPA (C22:5n‐6) was added to the synthesized lipid at sn‐2 position of the glycerol backbone, because the commercially available plasmalogen (C18:0p/C22:6n‐3) promoted only multilayer lamellar structures (data not shown), suggesting the important role of specific DPA (C22:5n‐6) fatty acid chains in CM formation. Of interest, we also examined whether plasmalogen‐carrying DPA (C22:5n‐6) or DPA fatty acid chains alone were sufficient to promote cubic phase transition *in vitro*.

**Fig. 6:**
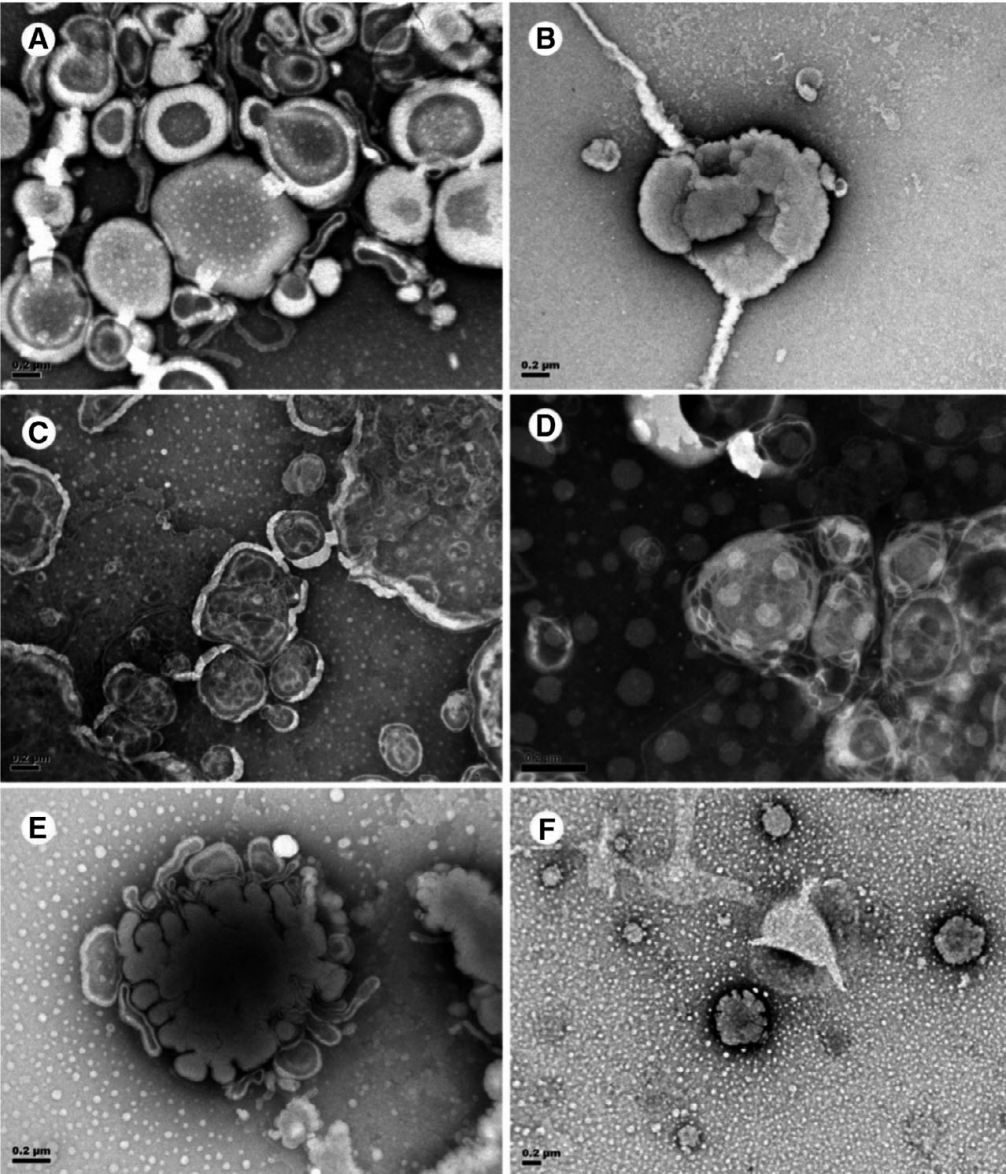
TEM micrographs of liposomes of lamellar phase lipids with or without plasmalogen modification. (A) Liposome construct (control) with a mixture of synthetic lipids; DOPC and DOPE form the lamellar lipid phase structure. (B) Liposome construct with DOPC, DOPE and diacyl‐PI (C22:5n‐6/C22:5n‐6). (C) Liposome construct with DOPC, DOPE and pPC (C16:0p/C22:5n‐6). (D) Liposome construct with DOPC, DOPE and pPE (C16:0p/C22:5n‐6). (E) Liposome construct with DOPC, DOPE and diacyl‐PI (C22:5n‐6/C22:5n‐6); pPC (C16:0p/C22:5n‐6); and pPE (C16:0p/C22:5n‐6). (F) A mixture of diacyl‐PI (C22:5n‐6/C22:5n‐6), pPC (C16:0p/C22:5n‐6) and pPE (C16:0p/C22:5n‐6). Scale bars: 0.2 µm. Three independent experiments were performed.

The control lamellar phase‐prone lipids were constructed as liposomes *in vitro* using DOPC and DOPE. The mixtures of DOPC and DOPE were used because they mimic the lipid components of the mitochondrial membranes; lamellar‐prone diacyl‐PC and non‐lamellar‐prone diacyl‐PE were the major phospholipids of mitochondrial membranes [22]. As indicated in Fig. 6A, vesicular multilamellar structures were formed by the mixture of DOPC–DOPE lipids. Incorporation of diacyl‐PI (C22:5n‐6/C22:5n‐6) to DOPC–DOPE mixture rendered coalescing of the lipids (Fig. 6B), suggesting that C22:5n‐6 fatty acid chains alone were probably not able to induce cubic transition. In contrast, pPC (C16:0p/C22:5n‐6) promoted the morphological changes on the lamellar liposomes (DOPC–DOPE).

Although from the negative staining TEM images alone, it is difficult to accurately depict the transformed morphology of lipids mixture (Fig. 6C,D). Whereas the morphological change is evidently convoluted, it is not highly ordered phase structures like classical hexagonal and/or cubic phases. Figure 6C,D illustrated the conversion of lamellar (DOPC–DOPE mixture) to reticular‐like network structure when pPC (C16:0p/C22:5n‐6) and pPE (C16:0p/C22:5n‐6) were added, respectively. Plasmalogen‐carrying DPA (C22:5n‐6) effectively curved the lamellar structures, and the two diacyl‐PI‐carrying DPA (C22:5n‐6/C22:5n‐6) fatty acid chains resulted in the coalescing of lipids formed by DOPC–DOPE mixture (Fig. 6B). The outcome shows a significantly different effect by adding diacyl‐PI (C22:5n‐6/C22:5n‐6) and appears uncanny. The next attempt led to the mixture of all three synthesized lipids (Fig. 5) with and without DOPC/DOPE. The mixture led to the lipids coalescing with significant curvatures, which also appear to be strange (Fig. 6E,F).

In support of our results, a recent parallel study adopted similar techniques; monoolein/DOPC nanostructured lipid phases with alteration of plasmalogens carrying DPA (C22:5n‐6) were used to validate the potency of plasmalogens on the membrane curvature and/or membrane rearrangements [7]. The results showed that plasmalogens‐carrying C22:5n‐6 fatty acids at sn‐2 position effectively curved the lamellar phase structures and induced multiple nanostructures such as inverted hexagonal (H_II_), double diamond cubic phase, double‐membrane vesicles and multilamellar whorl topologies, indicating the importance of DPA‐based PE and PC plasmalogens in inducing membrane curvature [7]. From our negative staining TEM results and the published data, plasmalogens appear to promote membrane fusion to form reticular‐like structures, suggesting their preference of promoting lamellar to nonlamellar membrane transformation.

It is arguable that although DOPC–DOPE mimics the major lipids of mitochondrial membranes, their innate morphology is lamellar, whereas CM formation *in vivo* suggests a morphological transition from tubular to CM [23]. Here, our observations propose that both plasmalogens and DPA (C22:5n‐6) may together play a key role in lipid membrane phase transition. More studies are required to further extrapolate our hypothesis that plasmalogens might work synergistically in modification of lipid membrane phase structures to higher‐ordered hexagonal or cubic morphologies when the role of these special plasmalogens (C16:0p/C22:5n‐6) is explored further in the future. With relevance to biological CM, it is highly possible that *in vivo*, lipids, proteins and other ionic milieu [24] or pH factors may together partake in a full CM transformation.

## Conclusions

This study provides the first clue toward understanding the potential role of plasmalogens supplementation in determining cell organellar membrane architecture. In particular, it relates to the availability and capability of the membrane plasmalogens to induce membrane curvature. Plasmalogens have been proposed to play an important role in membrane dynamics and trafficking [1, 7, 25, 26] and facilitating membrane fusion [27]. Several reports have shown that highly heterogeneous bilayer membranes enriched in plasmalogens are present in synaptic vesicles [1, 12, 28], which are involved in neurotransmitter release. Even a small amount of reduction in either the vinyl–ether content and/or the polyunsaturated fatty acid content of vesicles dramatically reduces the number of successful membrane fusion events [29].

As such, the data in this report may shed some light on the emerging structural property of plasmalogens capable of facilitating nonlamellar CM formation in amoeba *Chaos* cells, suggesting that these unique vinyl–ether‐bonded phospholipids may promote membrane fusion and/or vesicular formation and modulate membrane trafficking that are crucial in multiple cell processes, especially in neurons and neuroglial cells. Insights on such structural attributes of plasmalogens may also explain the reduced levels of plasmalogens in neurodegenerative diseases, including Alzheimer’s disease [13, 30, 31, 32, 33, 34], and also the dietary plasmalogen supplementations on potential cognitive improvements in patients with Alzheimer’s disease [35, 36].

## Conflicts of interest

The authors declare no conflict of interest.

## Author contributions

KC, ZAA and YD provided the conception of the paper. KC performed the experiments. KC, ZAA and YD analyzed and processed the data. KC and ZAA wrote the manuscript with input from RZ and YD. All authors critically read, edited and approved the manuscript.

## Data Availability

The analyzed datasets generated during this study are available from the corresponding author on request.
